# Effect of CPP/ACP on Initial Bioadhesion to Enamel and Dentin *In Situ*

**DOI:** 10.1155/2014/512682

**Published:** 2014-10-21

**Authors:** Susann Grychtol, Sabine Basche, Matthias Hannig, Christian Hannig

**Affiliations:** ^1^Clinic of Operative Dentistry, Faculty of Medicine Carl Gustav Carus, TU Dresden, Fetscherstraße 74, 01307 Dresden, Germany; ^2^Clinic of Operative Dentistry, Periodontology and Preventive Dentistry, University Hospital, Saarland University, Building 73, 66421 Homburg, Germany

## Abstract

The present *in situ* study investigated the influence of a preparation containing CPP/ACP (caseinphosphopeptide-amorphous calcium phosphate) (GC Tooth mousse) on initial bacterial colonization of enamel and dentin. Therefore, pellicle formation was performed *in situ* on bovine enamel and dentin specimens fixed to individual upper jaw splints worn by 8 subjects. After 1 min of pellicle formation GC Tooth mousse was used according to manufacturer's recommendations. Rinses with chlorhexidine served as positive controls. Specimens carried without any rinse served as negative controls. After 8 h overnight exposure of the splints, bacterial colonization was quantified by fluorescence microscopy (DAPI and BacLight live/dead staining). Additionally, the colony forming units (CFU) were determined after desorption. Furthermore, the effects on *Streptococcus mutans* bacteria were tested *in vitro* (BacLight). There was no significant impact of CPP/ACP on initial bacterial colonization proved with DAPI and BacLight. Determination of CFU showed statistical significance for CPP/ACP to reduce bacterial adherence on enamel. The *in vitro* investigation indicated no antimicrobial effects for CPP/ACP on *Streptococcus mutans* suspension. Under the chosen conditions, CPP/ACP (GC Tooth mousse) had no significant impact on initial biofilm formation on dental hard tissues. The tested preparation cannot be recommended for biofilm management.

## 1. Introduction

The prevalence of caries caused by adherence of bacteria and biofilm formation is still one of the greatest challenges in dentistry. Biomimetic and bioinspired oral health products containing nanoparticles have been developed to improve biofilm management [1, 2].

GC Tooth Mousse (Tooth Mousse, MI Paste; GC America) is a water based cream containing CPP/ACP. Caseinphosphopeptide- (CPP-) amorphous calcium phosphate (ACP) molecules (Recaldent) are derived from casein, a natural protein extracted from milk [3]. The caseinphosphopeptides stabilize nanoclusters of amorphous calcium phosphate (ACP) in a metastabile solution [3, 4]. The multifactorial anticariogenic mechanisms of CPP/ACP result from maintaining a supersaturated state of enamel minerals at the tooth surface [3]. CPP/ACP has already been shown to prevent demineralization and to promote remineralization of subsurface enamel lesions in animal and human* in situ* studies [5–7].

However, there are contradictory research findings about the anticariogenic effects of CPP/ACP. Morgan et al. investigated, using digital bitewing radiographs, approximal caries progression of 2.720 subjects [8]. One group chewed a gum containing CPP/ACP, whereas the other group chewed identical gum without CPP/ACP for 3 × 10 min/day over a period of 24 months. Finally, for the subjects who chewed the CPP/ACP gum caries progression was 18% less than for the CPP/ACP free gum group. In conclusion, CPP/ACP significantly reduced progression and enhanced regression of approximal caries [8]. A recent study by Sitthisettapong et al. came to a different result [9]. The double blind, placebo controlled clinical trial determined the effect of CPP/ACP paste on dental caries in primary teeth [9]. High-caries-risk children aged between 2.5 and 3.5 years received CPP/ACP paste (*n* = 150) in addition to regular tooth brushing with fluoridated toothpaste. The placebo control (*n* = 146) only used fluoridated toothpaste. The results were recorded with ICDAS after 6 months and one year. The clinical trial did not detect any difference between both groups. Enamel caries increment was recorded in both, the experimental and the control group. In conclusion, as compared with the fluoridated toothpaste, CPP/ACP had no additional caries preventive properties [9].

There is evidence that CPP/ACP nanocomplexes influence bacterial adhesion to the tooth surface both* in vitro* and* in situ *[10–12]. The effect of CPP/ACP on* Streptococcus mutans* biofilm formation has been investigated* in vitro* [13]. A decrease in the total number of adherent bacteria was detected on HA disks pretreated with CPP-ACP in combination with acidulated phosphate fluoride [13]. Former* in vitro* studies by Vacca-Smith et al. examined the effect of milk and casein on the adherence of* Streptococcus mutans* to saliva-coated hydroxyapatite [14]. They pointed out that kappa-Casein, when bound to sHA, inhibited streptococcal adherence to sHA* in vitro* [14]. Immunolocalization studies indicate the incorporation of CPP/ACP into plaque by binding to the surfaces of bacterial cells, to components of the intercellular plaque matrix, and to adsorbed proteins on the tooth surfaces, thereby possibly influencing the process of biofilm formation [1, 7, 12]. Furthermore, CPP/ACP might become incorporated into the pellicle layer in exchange for the streptococci-related receptors, thus inhibiting or modifying the bacterial adherence [12].

The effect of CPP-ACP on intraoral biofilm formation has also been determined in an* in situ* study using germanium surfaces as substrate for the investigation of bacterial biofilm formation [11]. This study showed that the presence of CPP/ACP agent delayed the biofilm formation considerably by changing the surface charges [11, 15]. However, little is known about the interaction of CPP/ACP with the* in vivo* pellicle layer on dental hard tissues. Particularly the impact of CPP/ACP on initial biofilm formation on dentin has not been considered yet. Therefore, the aim of this study was to investigate the antibacterial and antiadhesive properties of CPP/ACP using elaborate methods such as fluorescence microscopy [16, 17]. It was hypothesized that the tested preparation GC Tooth mousse containing CPP/ACP reduces initial bacterial colonization on enamel and dentin* in situ*.

## 2. Materials and Methods

### 2.1. Specimens and Subjects

Eight subjects (age: 24–42 years), all members of the laboratory staff, participated in the study. Visual oral examination was carried out by an experienced dentist. The subjects showed plaque indices and gingivitis indices close to zero and no untreated carious lesions. The salivary flow rate was physiological. The subjects gave their informed written consent to participate in the study. The study design has been approved by both the Medical Ethics Committee of the Medical Association of Saarland, Germany (# 18/10) and the Medical Ethics Committee of TU Dresden (# EK 147052013). Cylindrical enamel and dentin slabs (diameter 5 mm, surface area 19.63 mm^2^, and height 1.0 mm) were obtained from the facial surfaces of bovine incisors of BSE-negative 2-year-old cattle. For preparation of dentin specimens the enamel was removed and the outer dentin layer was used for the oral exposure experiments. The surfaces of all samples were polished by wet grinding with abrasive paper (400–4000 grit). Disinfection of the dentin samples was performed by ultrasonication in ethanol (70%) for 1 min, followed by air drying and 1 min ultrasonication in EDTA (3%) to remove the smear layer [17]. The slabs were washed twice for 5 min in distilled water. The enamel samples were treated by ultrasonication for 1 min in 3% NaOCl [18, 19]. Then they have been washed twice for 5 min in distilled water and disinfected by 10 min ultrasonication in ethanol (70%). All specimens were stored in distilled water for 24 h before exposure in the oral cavity.

### 2.2. Tested Preparation

GC Tooth mousse (Tooth Mousse, MI Paste; GC America, topical crème with bioavailable calcium und phosphate) was tested. A chlorhexidine-based mouth rinse (0.2% chlorhexidin-digluconate, meridol med CHX 0.2%, GABA, Lörrach, Germany) served as a reference.

### 2.3. BacLight Viability Assay,* In Vitro* Experiment

The LIVE/DEAD BacLight Bacterial Viability Kit (Invitrogen, Molecular probes, Darmstadt, Germany) adopts two stains—green fluorescent SYTO 9 stain (component A) and red-fluorescent propidium iodide stain (component B) [20]. The assays were performed according to manufacturer's instructions. The fluorescence intensity was measured in a 96-well plate reader. A suspension of* Streptococcus mutans* in saline solution was prepared after cultivation overnight; 50% of the bacteria were inactivated with heat (1 h; 95°C). Dilutions of Tooth mousse and chlorhexidine were prepared with saline solution (0.9% NaCl, sterile). The diluted preparations (CHX, Tooth mousse) were mixed 1 : 1 with vital bacteria and incubated for 10 min. Subsequently, these suspensions were mixed with heat-inactivated bacteria (0 : 100; 5 : 95; 25 : 75; 45 : 55; 50 : 50). A volume of 0.5 *μ*L of BacLight-staining solution (component A : B = 1 : 1) was added to 250 *μ*L of these mixtures. Afterwards, the staining-preparation was incubated for 10 min in a dark chamber. A volume of 100 *μ*L from each sample was pipetted twice in a microtiter plate, and the fluorescence was measured. The excitation wavelength was 470 nm; emission was recorded at 530 nm for the vital and at 620 nm for the avital cells. Duplicates were measured to equalize inhomogeneity of the suspension. For evaluation of the recorded data, the ratio of vital and dead/avital cells was calculated (ratio = emission vital/emission dead bacteria). Experiments with saline solution served as a reference.

#### 2.3.1. Oral Exposure of the Samples

For* in situ* bioadhesion, individual upper jaw splints were vacuum formed from 1.5 mm thick methacrylate foils. Cavities were prepared in the buccal aspects of the splints at the sites of the premolars and the 1st molar. The slabs were fixed on the splints using polyvinyl siloxane impression material (Provil novo light regular set, Heraeus Kulzer, Germany). Before oral exposure of the splints the subjects brushed their teeth without toothpaste and rinsed with tap-water. The splints were carried intraoral for 1 min to ensure pellicle formation on the surfaces. Thereafter the subjects applied 0.5 grams of GC Tooth mousse on the samples and left it undisturbed for 3 min in the oral cavity. Then the remaining GC Tooth mousse was spread throughout the mouth for further 2 min and finally spit out thoroughly. Rinses with chlorhexidine (8 mL for 1 min) were performed as positive controls; specimens carried without any rinse served as negative controls. Subsequently, the splints were kept in the oral cavity for another 8 h overnight. After oral exposure the slabs were removed immediately from the splints and thoroughly rinsed with running distilled water for 15 s to remove nonadsorbed salivary remnants. Six passes of the trial were performed per subject. The enamel and dentin slabs were tested for the amount of adherent bacteria with DAPI (4′,6-diamidino-2-phenylindole), live/dead staining (BacLight), and the CFU method (colony forming units), each one determined twofold.

#### 2.3.2. Fluorescence Microscopic Assays

The epifluorescence assays were conducted at 1,000-fold magnification as in several previous studies (Axioskop II, ZEISS, Oberkochen, Germany) [20]. The number of cells observed in 10 randomized microscopic ocular grid fields per sample was counted. The area of ocular grid fields (0.0156 mm²) allowed calculating the number of cells per square centimeter of the evaluated surface [16, 17].

### 2.4. DAPI Staining

DAPI (4′,6-diamidino-2-phenylindole) forms fluorescent complexes by binding to AT-rich regions of the double-stranded DNA [17, 21]. The staining was conducted as described previously [22]. First, the samples were rinsed with saline solution. For staining, the samples were covered with 1 mL DAPI working-solution (Merck, Darmstadt, Germany) and incubated in a dark chamber for 15 min at room temperature. The solution was then poured off and the specimens were covered with methanol and air-dried at room temperature. The slabs were stuck to a slide and analyzed by epifluorescence microscopy.

#### 2.4.1. BacLight Viability Assay,* In Situ* Experiments

The BacLight kit was adopted for visualization of vital and avital bacteria on enamel and dentin slabs after oral exposure. Similar amounts of component A (Syto9 1.67 mM/propidium iodide, 1.67 mM, 300 *μ*L DMSO) and B (Syto 9 dye, 1.67 mM/propidium iodide, 18.3 mM, 300 *μ*L DMSO) were mixed; 2 *μ*L was added to 1 mL of saline solution. The slabs were incubated in this solution for 10 min. Then the samples were rinsed with saline solution and examined by fluorescence microscopy with the fluorescein-diacetate filter (FDA) and the ethidium-bromide filter.

### 2.5. Colony-Forming Units

For determination of the colony-forming units (CFU), the specimens were vortexed for 2 min in sterile tubes with 1 mL 0.9% sodium chloride and for 1 min in an ultrasonic bath on ice. This solution was serially diluted up to 1 : 10^4^ in saline solution and plated on Columbia blood agar (CBA, aerobic bacteria and facultative anaerobic bacteria) or on yeast-cysteine-blood agar, respectively (HCB, anaerobic bacteria). The HCB plates were incubated for 7 days in anaerobic jars (Merck) at 37°C using BBL GasPak Anaerobic System Envelopes (Becton Dickinson, NJ, USA), the CBA plates under aerobic conditions with 5% CO_2_ for 2 days.

### 2.6. Statistics

Statistical evaluation was performed by means of the Kruskal-Wallis test and additional pair-wise comparison was carried out using the Mann-Whitney *U* test; *P* values were evaluated according to Bonferroni correction (*P* < 0.017). The Software used was SPSS 17.0 (IBM, Ehningen, Germany) [23, 24].

## 3. Results

**Figure 1:**
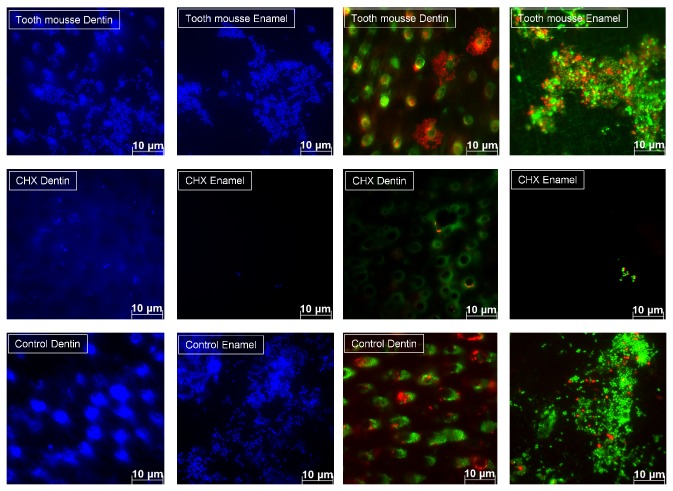
DAPI staining (blue) and live/dead staining (red/green): representative images of CPP/ACP samples and control-specimens (CHX, no rinse) after 8 h oral exposure. On enamel control samples vast areas were coated by bacterial aggregates or monolayers; dentin samples show a preferred colonization at the dentin tubules. Initial rinses of chlorhexidine reduced the bacterial adhesion considerably. CPP/ACP had no impact on the amount of adherent bacteria, as compared with unrinsed controls.

**Figure 2:**
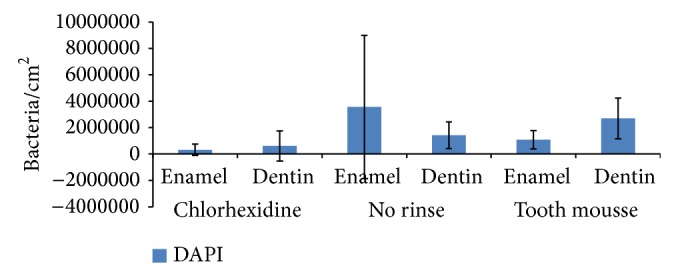
DAPI staining for detection of total amount of bacteria after application of GC Tooth mousse, rinse with chlorhexidine, or no rinse. After 1 min of pellicle formation, GC Tooth mousse was applied and rinses with chlorhexidine (8 mL/1 min) were performed, respectively. The enamel and dentin samples were carried overnight for 8 h* in situ* fixed on individual upper jaw splints. MV ± SD, *n* = 8 subjects, two samples per subject and subgroup.

**Figure 3:**
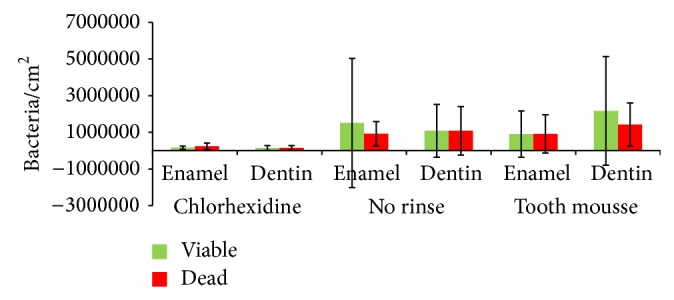
Live/dead staining (BacLight) for detection of viable and dead bacteria after application of GC Tooth mousse, rinses with chlorhexidine, or no rinse. Exposure of enamel and dentin samples for 8 h overnight* in situ*. MV ± SD, *n* = 8 subjects, two samples per subject and subgroup.

Initial microbial colonization was visualized with DAPI staining and BacLight (Figure 1). CPP/ACP had no effect on the amount of adherent bacteria, as compared with unrinsed controls (Figures 1, 2, and 3). After 8 h of oral exposure monolayers and chains of microorganisms were visible on the enamel slabs, covering more than 50% of the surface. The respective dentin slabs were colonized preferentially at the orifices of the dentin tubules. After application of Tooth mousse no modification of bacterial adherence is visible, neither on the enamel nor on the dentin samples. Both quantification and viability of the microorganisms were not significantly affected by CPP/ACP (Figures 1, 2, and 3, Table 1). As compared with controls, chlorhexidine had considerable effects on the amount of adherent bacteria on enamel and dentin samples (Figures 1, 2, and 3, Table 1). Adherent bacteria on the enamel and dentin slabs were quantified with DAPI and live/dead staining (Figures 2 and 3). According to DAPI-staining, there was no significant impact of CPP/ACP on the initial bacterial colonization on enamel (1.08 × 10^6^ ± 6.87 × 10^5^/cm^2^) and dentin samples (2.70 × 10^6^ ± 1.55 × 10^6^/cm^2^) as compared with unrinsed controls (enamel: 3.57 × 10^6^ ± 5.41 × 10^6^/cm^2^; dentin: 1.43 × 10^6^ ± 1.01 × 10^6^/cm^2^). All methods showed a high interindividual and intraindividual variability. A significant effect was observed for chlorhexidine, as expected (enamel: 3.19 × 10^5^ ± 4.35 × 10^5^ bacteria/cm^2^, dentin: 6.11 × 10^5^ ± 1.14 × 10^6^ bacteria/cm^2^).

**Figure 4:**
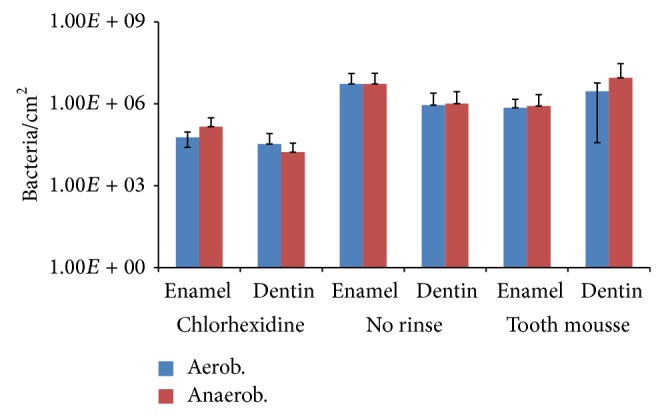
Quantification of colony forming units (CFU) after desorption for determination of adherent aerobic and anaerobic bacteria. Exposure of the specimens at the buccal sites of the upper 1st and 2nd premolar and 1st molar for 8 h overnight MV ± SD, *n* = 8 subjects, 2 samples per subject and preparation.

The determination of the colony forming units showed significant influence of CPP/ACP on initial bacterial colonization on enamel samples (Figure 4, Table 1). The CFU data for aerobic and anaerobic bacteria showed a high variability. Nevertheless, statistical evaluation by Kruskal-Wallis test and Mann-Whitney *U* test indicated a significant impact of chlorhexidine rinses on the amount of culturable bacteria (Table 1).

**Figure 5:**
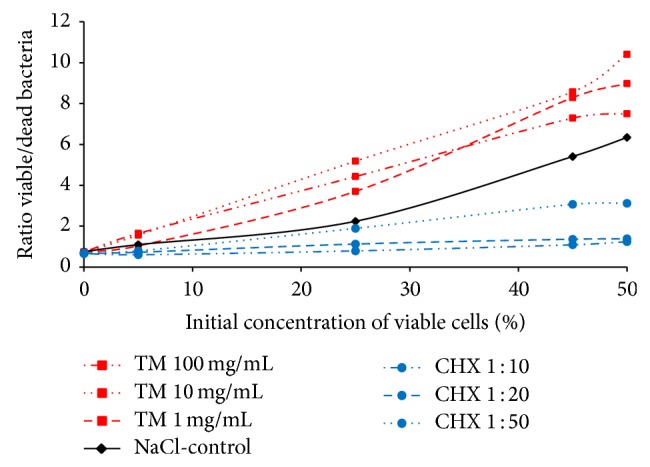
Effect of CPP/ACP (red), chlorhexidine (CHX) (blue), and saline solution (control) (black) on the viability of* S. mutans in vitro*. A suspension of* S. mutans* was incubated with CPP/ACP and chlorhexidine solutions in different concentrations. After incubation, the samples were admixed to proportions of the basic suspension. The amount of vital and dead bacteria was determined by BacLight bacterial viability assay. The measurements were carried out in duplicate and the ratio was calculated: emission 530 nm and emission 620 nm are representing emission vital and emission dead bacteria, respectively.

The ratio of viable and dead bacteria amounted to 1.64 for enamel and to 1.00 for dentin in the unrinsed control group. It was decreased by the application of chlorhexidine on enamel (0.67) and remained unchanged on dentin (0.98). CPP/ACP also showed a decline of the ratio on enamel (0.98), but an increase on dentin (1.52); that is, there were higher proportions of viable bacteria. The* in vitro* experiments with suspensions of* Streptococcus mutans* indicated no antibacterial effects for CPP/ACP (Figure 5). As expected, the gold standard chlorhexidine showed antibacterial properties in a dose-dependent manner (Figure 5).

## 4. Discussion

For the first time, the present* in situ* study investigated the influence of a customary oral care product containing CPP/ACP (Tooth Mousse, MI Paste; GC America) on the initial bacterial colonization of enamel and dentin. Our study revealed no antibacterial and antiadherent effects for CPP/ACP. The bioadhesion processes were determined* in situ*, considering that* in vivo* and* in vitro* conditions differ considerably [25]. The methodical approach, including bovine enamel and dentin specimens fixed to individual upper jaw splints, has already been used in previous studies [20, 25]. The overnight trial is an approved method to evaluate the impact of oral therapeutics of initial biofilm formation [25]. During this time ingestion, potation and other external influences can be excluded. It has to be considered that the application of the tested preparation GC Tooth mousse was performed with paste, whereas chlorhexidine rinses served as controls. Accordingly, a direct comparability was not ensured. Yet, both application types were performed in accordance with the manufacturer's recommendations. After the application of GC Tooth mousse or rinses with chlorhexidine the splints were carried for 8 h overnight. The chlorhexidine mouth rinse is considered as gold standard in plaque reducing properties and served as positive control [26]. Samples carried without any rinse served as negative controls. This technique allows the investigation of initial bacterial colonization with the greatest possible standardization under* in situ* conditions. Despite these precautionary measures, high interindividual and intraindividual variability was observed. This drawback seems to be characteristic for initial bioadhesion in the oral cavity and corresponds to previous investigations, for example, recently published pilot studies of edible oils or Biorepair [17, 20, 22]. Another limitation of the study is the small number of subjects. It has to be kept in mind when interpreting the data. However, the aim of this* in situ* study was to gain insight into the principal effect of a preparation containing CPP/ACP on initial biofilm formation. DAPI and live/dead staining demonstrated the lacking effect of CPP/ACP on both quantification and viability of the adherent bacteria. Statistical evaluation pointed out that there was no significant influence of CPP/ACP on initial bacterial colonization after 8 h* in situ* exposure of the samples (Table 1). The CFU method showed a pronounced variability and significant impact of GC Tooth mousse on initial biofilm formation on enamel samples. However, with the culture plate method based on desorption and ultrasonication only 50% of the bacterial strains can be cultivated, whereas fluorescence microscopic methods (DAPI, BacLight) investigate the bacteria in the adherent state [27].

Former* in vitro* investigations indicated that CPP/ACP may affect bacterial adhesion at the tooth surface [10]. Schüpbach et al. visualized the incorporation of CPP into the salivary pellicle with SEM und TEM, with reduction of the adhesion of mutans streptococci [12]. So far,* in situ* studies regarding the effect of CPP/ACP on bacterial adherence are confined to investigations of plaque samples [28]. In the present* in situ* study with enamel and dentin samples the investigation of bioadhesion took place in a standardized manner. Although bovine dentin tubes have a greater diameter than those in human dentin, and there are more tubules/cm^2^, human and bovine dentin is alike and biofilm formation on these surfaces is similar [25, 29].

The missing effect of CPP/ACP on bacterial adhesion to the dental hard tissues is contradictory to previous* in vitro* and* in situ* studies [10–12]. It was suggested that the adsorption of CPP/ACP on enamel could cause an increase in the surface net negative charge, thereby influencing the long-range interactions with microbes through the development of repulsive forces [15, 30]. Furthermore CPP/ACP might modify long-term adhesion by masking the streptococci-related receptors on salivary molecules [31]. Bacterial receptors might be blocked;coaggregation and bacterial adhesion to the pellicle might be reduced [10, 32, 33]. On the one hand the lacking influence of CPP-ACP on initial bacterial colonization might be caused by the masking effect of the pellicle. Otherwise a slight local stability of CPP-ACP at the tooth surface is conceivable.

Rose demonstrated* in vitro* that CPP/ACP and calcium compete for the same binding sites on* S. mutans*. It was confirmed that CPP/ACP binds with twice the affinity to bacterial cells compared to calcium [10]. The therefore enhanced availability of calcium in the plaque has anticaries protective effects by promoting remineralization and hampering demineralization. Furthermore, there is evidence that a high concentration of free calcium may have bactericidal or bacteriostatic effects [10]. These investigations are limited to* Streptococcus pneumonia* and* Streptococcus faecalis* [34, 35]. Direct antibacterial effects of CPP/ACP on suspensions of* S. mutans* were investigated in our* in vitro* experiment. There were no antibacterial effects for CPP/ACP on* S. mutans* detectable. This was to be expected: antibacterial characteristics for caseine, calcium, or phosphate on* Streptococcus mutans* have not been observed, yet.

In general, the efficacy of CPP-ACP in oral biofilm management is doubtful. As aforementioned, there are contradictory results for the effects of CPP/ACP* in vivo* and* in situ*. Beside our investigations the potential of CPP-ACP to remineralize subsurface enamel lesions was often provided with the use of CPP-ACP chewing gums [7]. However, Schirrmeister et al. found no significant differences between chewing gums containing calcium and calcium-free chewing gums with regard to change of mineral loss and lesion depth of enamel samples [36]. Referring to the impact of CPP-ACP on dentin hypersensitivity different findings were achieved as well. Kowalczyk et al. revealed insufficient effectiveness and short-term therapeutic effects in treating hypersensitivity of dentine using GC Tooth mousse [37]. A recent* in situ* study showed on the contrary that CPP-ACP combined with fluoride is slightly effective in reducing dentinal hypersensitivity [38].

## 5. Conclusions

Under the conditions chosen in the present* in situ* study the tested preparation containing CPP/ACP (Tooth Mousse, MI Paste; GC America) has no significant impact on the initial bacterial colonization on enamel and dentin. Thus, it cannot be recommended for biofilm management.

## Figures and Tables

**Table 1:** Statistical evaluation of the *in situ* experiments with the different preparations; Kruskal-Wallis test and additional pairwise comparison with Mann-Whitney *U* test (*P* < 0.05).

Kruskal-Wallis test: *P* < 0.001	Enamel		Dentin		
	Tooth mousse	Chlorhexidine	No rinse	Tooth mousse	Chlorhexidine
*DAPI *					
Enamel					
No rinse	n.s.	<0.001	n.s.	n.s.	n.s.
Tooth mousse		0.001	n.s.	<0.001	n.s.
Chlorhexidine			<0.001	<0.001	n.s.
Dentin					
No rinse				0.008	0.001
Tooth mousse					<0.001

*BacLight viable bacteria *					
Enamel					
No rinse	n.s.	n.s.	n.s.	n.s.	n.s.
Tooth mousse		n.s.	n.s.	n.s.	n.s.
Chlorhexidine			<0.001	<0.001	n.s.
Dentin					
No rinse				n.s.	<0.001
Tooth mousse					<0.001

*BacLight dead bacteria *					
Enamel					
No rinse	n.s.	0.001	n.s.	n.s.	<0.001
Tooth mousse		0.012	n.s.	n.s.	<0.001
Chlorhexidine			n.s.	<0.001	n.s.
Dentin					
No rinse				n.s.	<0.001
Tooth mousse					<0.001

*CFU: aerobic *					
Enamel	0.007	<0.001	0.001	n.s.	<0.001
No rinse		<0.001	n.s.	n.s.	<0.001
Tooth mousse			n.s.	<0.001	n.s.
Chlorhexidine					
Dentin					
No rinse				0.004	<0.001
Tooth mousse					<0.001

*CFU: anaerobic *					
Enamel					
No rinse	0.011	<0.001	n.s.	n.s.	<0.001
Tooth mousse		0.008	n.s.	<0.001	<0.001
Chlorhexidine			n.s.	<0.001	n.s.
Dentin					
No rinse				<0.001	<0.001
Tooth mousse					<0.001
